# Evaluation of the effects of COVID-19 on pregnancy, fetus and newborn, and treatment management

**DOI:** 10.14744/nci.2021.45577

**Published:** 2022-02-11

**Authors:** Senol Comoglu, Sinan Ozturk, Merve Caglar Ozer, Baris Ertunc

**Affiliations:** 1 Department of Infectious Diseases and Clinical Microbiology, Umraniye Training and Research Hospital, Istanbul, Turkey; 2 Department of Infectious Diseases and Clinical Microbiology, Trabzon Kanuni Training and Research Hospital, Trabzon, Turkey

**Keywords:** COVID-19, newborn, pregnancy

## Abstract

**Objective:**

During pregnancy, changes occur in many systems, including the immune system. In line with our experience in the previous years, COVID-19 infections have negative effects on pregnancy. In our study, it was aimed to evaluate the effects of COVID-19 on pregnancy, fetus and newborn, and treatment management.

**Methods:**

In our study, 63 patients followed up between April 1, 2020 and April 1, 2021, were evaluated. Demographic data, symptoms, laboratory data, treatments, clinical course and delivery characteristics of the patients, as well as pathologies in the fetus and newborn were investigated retrospectively. The obtained data were statistically analyzed with Statistical Package for the Social Sciences.

**Results:**

In this study, 63 pregnant COVID-19 patients aged 19–37 years were included in the study. Fifty of the patients had symptoms of COVID-19 at the time of admission. At the time of admission, 13 patients required oxygen, and ten of these patients had severe radiological involvement. Seven patients were admitted to the intensive care unit, and three of them required invasive mechanical ventilation and deceased afterward. All newborns were found negative for the COVID-19 polymerase chain reaction test. Low birth weight has been detected in eight newborns and low Apgar score in 2 of them. Respiratory distress was observed in four newborns and they were discharged from intensive.

**Conclusion:**

Pregnant women have more disadvantages in the course of COVID-19 and have worse maternal outcomes. In addition, treatments such as Lopinavir/Ritonavir and hydroxychloroquine did not have any effect. These patients should be carefully evaluated and followed up.

**P**hysiologically, important changes occur in many systems such as the immune system, respiratory system, cardiovascular system, and coagulation functions during pregnancy. In the light of clinical experience obtained from pregnant women infected with COVID-19, it is thought that pregnant women are potentially vulnerable to serious COVID-19 infections [1]. The effects of COVID-19 on fetal growth and development, delivery, and neonatal health during pregnancy have not yet been clearly defined. Therefore, studies are needed to determine the possible effects of COVID-19 on the fetus and pregnancy. Another important problem in pregnant women who are experiencing COVID-19 asymptomatically or symptomatically is the quality of health services to be applied to these patients and how the treatment management should be carried out efficiently.

In this study, it was aimed to evaluate the effects of COVID-19 on pregnancy, fetus and newborn, and treatment management.

## Materials and Methods

In this study, 63 pregnant patients with the diagnosis of COVID-19 between April 1, 2020 and April 1, 2021, have been retrospectively analyzed. The research was conducted according to the COVID-19 guidelines of the Ministry of Health and the World Health Organization [2, 3]. The study has been approved by the Health Sciences University, Umraniye Training and Research Hospital Clinical Research Ethics Committee at November 18, 2021, protocol number 54132726–000–22135.

The diagnosis of COVID-19 was confirmed by polymerase chain reaction (PCR) testing. In addition to demographic and clinical data such as age, gestational week, fetal ultrasonography findings, cough, fever, headache, shortness of breath, weakness, myalgia, sore throat, smell and taste disorders, hemogram, C-reactive protein (CRP), and lactate dehydrogenase (LDH) and D-Dimer results were recorded on the study forms.

After the diagnosis, the pregnancy status and medical problems of the patients until delivery were obtained from the hospital registry system. Pre-eclampsia related to pregnancy, venous thromboembolism, hospitalization, admission to intensive care unit (ICU), need for mechanical ventilation, additional oxygen requirement, length of hospital stay, premature birth (delivery before 37 weeks of gestation), mode of delivery (cesarean or vaginal delivery), intrauterine fetal death and chorioamnionitis, and maternal death information were evaluated [4].

In the evaluation of the newborn, conditions such as birth weight, respiratory distress syndrome, hospitalization in the neonatal ICU, Apgar scores at the 1^st^ and 5^th^ minutes, enterocolitis, length of hospital stay, and neonatal death were analyzed. Treatment management included oxygen therapy, antiviral therapies, hydroxychloroquine, antibiotics, bronchodilators, mechanical ventilation, steroids, and admission to the ICU.

### Statistical Analysis

The data were analysed by the Statistical Package for the Social Sciences (IBM SPSS 26, New Orchard Road Armonk, Newyork 10504-12722 United States) program and descriptive statistical analysis was performed. The compatibility of the data obtained with the measurement with the normal distribution was made with the Kolmogorov–Smirnov test. Student’s t-test was used for data with normal distribution, and Mann–Whitney U test was used for data not with normal distribution. Chi-square test was used for the analysis of categorical variables. The data obtained by the measurement were expressed as the mean±standard deviation. The data obtained by counting were expressed as numbers (%). P<0.05 was considered statistically significant.

**Highlight key points**

• Due to the physiological changes in the immune system and many other systems in pregnant women, COVID-19 infection may cause more severe symptoms and complications in pregnant women.
• Lopinavir/ritonavir, hydroxychloroquine treatments were found to be ineffective in pregnant women infected with COVID-19.
• Oxygen need was found to be significantly higher in pregnant women with COVID-19 who were in the third trimester than those who were not.

## Results

A total of 63 pregnant patients between the ages of 19 and 37 were included in this study. The mean age of the patients was 28.78±4.61 years. Weeks of gestation at the time of admission were between 6 and 39 weeks, and the mean week of gestation was 27.4±8.9. A majority of the patients (n=47) were followed up in hospital and 16 of them were isolated at home. While 50 (79.4%) of the patients had symptoms at the time of admission, 13 (20.6%) had no symptoms. Cough was observed in 26 (41.3%) of our patients, myalgia in 25 (39.7%), fatigue in 22 (34.9%), shortness of breath in 21 (33.3%), fever in 13 (20.6%), headache in 12 (19%), sore throat in 6 (9.5%), loss of taste and smell in 3 (4.8%), and diarrhea in 2 (3.2%) individuals (Table 1). Six of the patients (9.5%) had comorbid diseases such as diabetes mellitus, hypertension, coronary artery disease, asthma, and chronic kidney failure. Two pregnant women were diagnosed with preeclampsia.

**Table 1.** Admission symptoms

Symptoms	Percent
Caugh	41.30
Myalgia	39.70
Fatigue	34.90
Shortness of breath	33.30
Fever	20.60
Headache	19
Sorethoat	9.50
Loss of taste/smell	4.80
Diahrerria	3.20

Thorax computed tomography (CT) scans have been performed in 17 patients on day 6.88±2.62 of symptoms revealed severe involvement in 10 (58.8%), moderate involvement in 3 (17.6%), mild involvement in 3 (17.6%), and no involvement 1 (5.6%) patient. Lymphopenia (lymphocyte <800/μL) was detected at the time of admission in 13 (20.3%) of the patients. The mean lymphocyte values of the patients at the time of admission were 1065±560.87/μL (230-2150), CRP 39.5±49.38 mg/L (1–197), D dimer 1541.9±1070.13 ng/ml (430–5599), and LDH 255.9±112.34 U /L (84–735).

A total of 36 (57.1%) patients received symptomatic treatment and 27 (42.9%) subjects specific treatment. The utilized agents were as follows: 12 (44.4%) hydroxychloroquine, 7 (25.9%) azithromycin, 17 (63%) lopinavir/ritonavir, and 11 (40.7%) steroid treatment. Anticytokine, plasmapheresis, and immunoglobulin were not used in any of the pregnant cases. Of the patients, 13 (20.6%) required oxygen, 7 (6.3%) needed intensive care, and 3 (4.8%) of them invasive mechanical ventilation. All of the pregnant women who underwent invasive mechanical ventilation were deceased.

No fetal ultrasonographic anomaly was detected in any of the 63 pregnant women and all of them had normal vaginal delivery or cesarean section (C/S). Ten patients gave birth in the acute phase of COVID-19 infection, five of these patients delivered prematurely. Of the pregnant women who gave birth, 40 (63.5%) had normal vaginal delivery and 23 (36.5%) had C/S delivery. Apart from gynecological indications, the delivery method has been decided according to the clinical feature of COVID-19 infection, whether in acute period or not. Three pregnant patients were taken to early C/S due to tachypneic and impaired hemodynamics and were deceased. Subjects who gave birth in the acute period of COVID-19 infection were followed up for an average of 15.1±8.3 days after delivery. Postpartum bleeding was observed in one of our patients and wound infection was observed in one of our patients (Table 2).

**Table 2.** Complications

Complications	Percent
Premature delivery	7.93
Postpartum bleeding	1.58
Wound ınfection	1.58
Low birth weight	12.69
Low Apgar	3.17
Respiratory distress of newborn	6.34

All newborns had negative COVID-19 PCR test. Eight newborns had low birth weight, two had low Apgar score, while four newborns had respiratory distress and were discharged after intensive care follow-up (Table 2).

Of the 13 patients who needed oxygen, seven were admitted to the ICU and three of them were intubated. While lymphocyte value was lower in those with oxygen requirement (p=0.002), CRP (p=0.001) and LDH values (p=0.002) were higher (Table 3). There was no difference between the D-dimer values of those who needed oxygen compared to those who did not (p=0.898). There was no difference between those with and without comorbid disease in terms of oxygen demand (p=0.419) and severity of involvement in thorax CT (p=0.863). Two of the three deceased patients had no comorbid disease. Most of the cases (63.5%) were in the third trimester and oxygen requirement was significantly higher in this period (p=0.015). There was no difference between the pregnant women in this group in terms of thorax CT involvement, presence of symptoms and need for intensive care (p>0.05).

**Table 3.** Relationship between mean laboratory parameters and patients who need oxygen

	Patients who need oxygen		
	Yes	No	p
Lymphocyte count at the admission	875.38 (294.268)	1388.39 (568.404)	0.002
Leastest lymphocyte count	678.33 (230.605)	1190.54 (461.26)	0.001
CRP at the admission	85.25 (65.333)	27.36 (36.259)	0.001
Hightest CPR Level	140.92 (85.259)	51.57 (51.245)	0.001

*: Numbers are indicated mean parameters and parantheses indicates standart deviations; CRP: C-reactive protein.

## Discussion

As a result of our clinical experience we have gained so far, it was seen that pregnancy itself does not pose an additional risk for the acquisition of COVID-19 infection, but that there were differences in the course of the infection compared to those who are not pregnant [5]. In a systematic meta-analysis conducted by Allotey et al. (2019) [6], it was reported that approximately 74% of pregnant women were asymptomatic. In this study, we have found that only 20.6% of the patients were asymptomatic. The reason for the low rate of asymptomatic patients in our study may be due to the selection of the cases admitted to the hospital. It should be evaluated in a wider population by including pregnant women who did not apply to the hospital.

Mild or moderate cold/flu-like symptoms have been reported in most symptomatic pregnant women [7]. Similarly, in this study, we have observed that non-specific symptoms were seen in half of the patients and serious symptoms such as oxygen requirement were observed in a minority of pregnant women. Thirteen of our 21 patients who described respiratory distress needed oxygen. In other patients, it was thought that the cause of dyspnea was mainly due to pregnancy.

In a prospective cohort study of Afshar et al. (2019) [8] on pregnant women, it was reported that the most common initial symptoms were cough, sore throat, myalgia, and fever in United States. Similarly, in this study, we have observed that the most common symptoms were non-specific complaints such as cough, myalgia, and fatigue.

Severe COVID-19 cases, such as the need for ICU admission, is relatively rare in women of reproductive age [9]. Jerings et al. (2021) [10] published an article indicating that COVID-19 was detected in 6380 (1.6%) of 406,446 pregnant women. Their retrospective analysis has approximately covered 20% of the American population of the data of patients hospitalized for delivery. They have emphasized that, although maternal death is rare among pregnant women followed up in the hospital, the mortality rate in pregnant women with COVID-19 (141 out of 100,000) was higher (5 out of 100,000) than in pregnant women without COVID-19. In our study, three of the patients have deceased and this could be explained with the fact that those patients have applied to hospital with severe symptoms. More reliable data on the maternal mortality rate due to COVID-19 can be obtained in population screenings that will be performed regardless of clinical symptoms.

Hantoushzadeh et al. (2020) [11] reported that seven of nine pregnant women with COVID-19 infection died due to COVID-19. They stated that there was no underlying health problem in five of the seven maternal deaths and that pregnancy may pose women at a higher risk for serious consequences of COVID-19 infection.

Knight et al. (2021) [12] have evaluated 427 pregnant women admitted to the hospital with COVID-19 infection. They elaborated that severe disease was more common in the later stages of pregnancy. In this study, it was identifed that 83% of symptomatic pregnant women were diagnosed at or after 28 weeks, and 52% were diagnosed at or after 37 weeks. In our study 63.5% of the pregnant women included were in the third trimester. Although, the oxygen requirement was significantly higher in pregnant women in the third there was no difference in the presence of symptoms.

DeBolt et al. (2020) [13] published that the probability of being admitted to the ICU for pregnant women with COVID-19 was found to be significantly higher than non-pregnant women (17.5% vs. 39.5%, p<0.01). In another study conducted in France, it was reported that the need for oxygen therapy, hospitalization in the ICU, and endotracheal intubation were higher in pregnant women than in non-pregnant women [14]. In our study, 20.6% of the patients required oxygen, 11.1% needed intensive care, and 4.8% invasive mechanical ventilation.

Lopinavir-ritonavir, which is used as a protease inhibitor in patients with acquired immunodeficiency syndrome, is an antiretroviral drug that is thought to be used in the treatment of SARS-COV-2 due to its pharmacokinetic properties. However, its use is not recommended because the plasma concentration may be below the levels to inhibit the replication of SARS-COV-2 in the studies conducted, the clinical benefit could not be shown in large randomized patients, and its effectiveness on mortality in hospitalized patients was not demonstrated [15, 16]. It was thought that hydroxychloroquine, which can be used in the treatment of antimalarial and autoimmune diseases, may be effective in the treatment because of its inhibition of SARS-COV-2 fusion into the host cell and its immunomodulatory effects. However, in randomized controlled studies conducted with both hospitalized and outpatients, it was observed that hydroxychloroquine had no effect on survival and length of hospital stay [15]. In our study, it was observed that lopinavir/ritonavir and hydroxychloroquine treatments were not effective in pregnant women infected with COVID-19.

### Conclusion

As a result, in the light of previous studies, it is seen that pregnant women have more disadvantaged in the course of COVID-19. COVID-19 has worse overall maternal outcomes, including an increased risk of death. For this reason, it should be kept in mind that the rates of ICU admission and mortality in pregnant patients are higher in many studies compared to non-pregnant patients, and these patients should be carefully evaluated and followed up, considering that the infection can progress severely.
